# Fibrin Glue in Postlaryngectomy Fistula-A Case Report 

**DOI:** 10.22038/ijorl.2019.38563.2366

**Published:** 2020-03

**Authors:** Hassan Vossoughinia, Mohammad Ali Zarringhalam, Daryosh Hamidi Alamdari, Ahmad Mehrdad Zinkanlou

**Affiliations:** 1 *Department of Gastroenterology and Hepatology ,Mashhad University of Medical Sciences, Mashhad, Iran.*; 2 *Departments of Otorhinolaryngology, Mashhad University of Medical Sciences, Mashhad, Iran.*; 3 *Stem Cell and Regenerative Medicine Research Group, Department of Biochemistry, Stem Cell Laboratory, Faculty of Medicine, Mashhad University of Medical Sciences, Mashhad, Iran.*

**Keywords:** Fibrin glue, Laryngectomy, Pharyngocutaneous Fistula

## Abstract

**Introduction:**

One of the most common complications following total laryngectomy is pharyngocutaneous fistula (PCF). Various methods have been proposed to treat this disorder in recent studies, including a range of simple and conservative treatments to more aggressive therapies, such as various surgical procedures. One of the most innovative and least developed methods is the use of plasma-rich compounds, such as fibrin glue.

**Case Report:**

The patient was a 55-year-old woman with a transglottic squamous cell carcinoma of the T3N0M0 stage and PCF development following total laryngectomy surgery with total thyroidectomy and bilateral elective cervical lymph node dissection level I-IV. In spite of conservative treatment, the fistula was not recovered after 3 weeks. It was decided to perform fibrin glue injection into the fistula tract via the endoscopic approach. One month after the fibrin glue injection, no evidence of contrast extravasation was observed on barium swallow test, and the fistula was completely closed.

**Conclusion:**

No PCF has been treated with fibrin glue using only the endoscopic technique. The present study showed that fibrin glue can be used as an effective way to treat chronic fistulas in head and neck surgeries.

## Introduction

Laryngeal cancer accounts for about 1% to 2% of all cancers and more than 20% of head and neck cancers (1). One of the most important surgical procedures for the treatment of laryngeal cancer is total laryngectomy, which is often used as an effective treatment; however, similar to other surgical procedures, it includes various complications. According to the literature, the incidence of complications following laryngeal cancer varies between 40-92% (1-5). One of the most common and difficult complications is pharyngocutaneous fistula (PCF). The PCFs occur following the disruption of neopharynx repair and salivary leakage. The incidence rate of this complication has been reported in many articles ranging from 3-65%; nevertheless, most of the studies have announced an incidence rate between 10-40% (6). There is still no general agreement on any of the introduced risk factors regarding this condition as independent factors. 

Anemia and low serum albumin levels before the surgery, comorbidity of patient (6-10,11), hypothyroidism, high tumor stage, previous tracheostomy, wide neck dissection (6,11-16), positive surgical margins (17,18), neopharynx reconstruction technique )T shape closure has the minimal risk of fistula) (7,19-21), and surgeon's experience are common risk factors; however, chemoradiotherapy before the surgery is probably the most important independent risk factor (3,4,7,12,16,22-24). Regardless of the risk factors and causes of this disorder, the incidence and treatment of PCF are among major and complicated problems with a high risk of morbidity, delay in starting adjuvant treatment, long-term hospitalization, and high costs, which require much more patience not only on the surgeon's side but also the patient and his family (19).Various methods have been proposed for the treatment of this complication in recent articles, including a range of simple and conservative treatments to more aggressive therapies, such as various surgical procedures. One of the most innovative and least developed methods is the use of plasma-rich compounds, such as fibrin glue. The purpose of the present study was to introduce a case of PCF following total laryngectomy and fistula treatment using endoscopic fibrin glue administration and subsequent review of the related literature.

## Case Report

The patient was a 55-year-old woman with a history of smoking and prolonged opium inhalation, referred to Ghaem clinic in Mashhad with dysphonia, hoarseness, and occasional aspiration since last year. No sign of odynodysphagia, stridor, and dyspnea was observed in the case.

**Fig 1:**
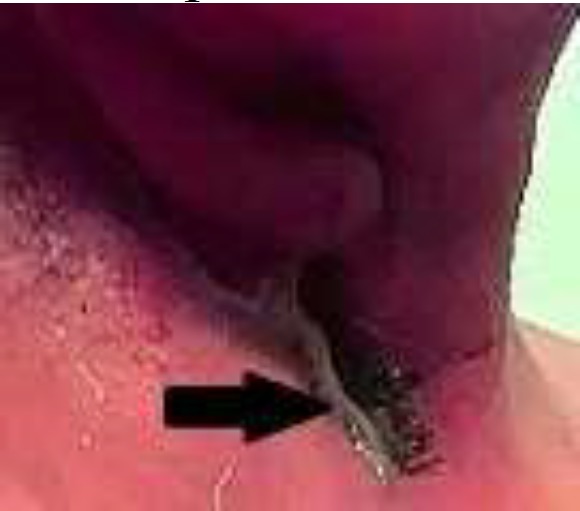
(A) The clinical view of Pharyngocutaneous Fistula in our patient. (B) Contrast extravasation from the neopharynx in computed tomography scan of the neck following total laryngectomy indicating the formation of salivary fistula

The patient did not previously undergo any treatment. In laryngoscopy, prior to surgery, left arytenoid, aryepiglottic fold, left false vocal cord, true vocal cord, anterior commissure, and subglottic region were tumoral. In addition, other parts of the larynx and hypopharynx were normal. The left vocal cord was fixed. In the preoperative computed tomography scan, there was a transglottic involvement on the left side, and cervical lymphadenopathy was not detected. Laryngeal cartilage and hyoid bone were reported to be normal. The right paraglottic space was involved, and the epiglottis was normal. The tongue, esophagus, trachea, and thyroid were normal. The biopsy of the affected areas represented the well-differentiated squamous cell carcinoma. Furthermore, chest X-rays and liver function tests were normal. The patient was ultimately diagnosed with a transglottic squamous cell carcinoma of the T3N0M0 stage. Moreover, total laryngectomy with total thyroidectomy (due to subglottic involvement) and bilateral elective cervical lymph node dissection level I-IV were performed. The tumor was macroscopically removed, and the surgical margin was checked with a frozen section for the presence of the tumor. The neopharynx was closed with the suturing technique. The patient diagnosed with salivary fistula was initially treated with conservative management, including total parenteral nutrition, pressure dressing, and broad-spectrum antibiotic therapy (Fig.1).

Despite the use of broad-spectrum antibiotics in the 12^th^ day, due to swelling and cervical erythema on the right side, the case with a diagnosis of infectious colonization, followed by the salivary fistula, was transferred to the operating room.

In addition, the drainage and diversion of salivary secretion away from the carotid sheath were performed under general anesthesia. Moreover, in order to sterilize the fistula and help the formation of fibrosis to close it, 10 cc of the acetic acid solution diluted to 0.25% was orally prescribed three times a day. 

To reduce the flow of saliva botulinum toxin injections in both submandibular glands were conducted under ultrasound guidance, and propantheline (i.e., an anticholinergic drug) was simultaneously prescribed for the patient. 

In spite of conservative treatment, the fistula was not recovered after 3 weeks.

Due to the probable surgical procedures, such as gastric pull-up or free flap reconstruction, in the final stages of fistula treatment failure, the jejunostomy was inserted instead of gastrostomy, and intestinal gavage was initiated. There was no evidence of positive surgical margins on permanent specimen pathology. 

Regarding the completion of the medication, conservative treatment, lack of response to the closure of the fistula, and risk of carotid injury with the salivary flow, it was considered to repair fistula by a free flap or gastric pull-up. Therefore, a thoracic surgeon was consulted about gastric pull-up considering the fistula repair.

It was decided to perform fibrin glue injection into fistula tract via the endoscopic approach after consultation with a gastroenterologist and thorax surgeon and according to the successful management of fistula with the use of plasma-rich compounds, such as fibrin glue, in addition to patient's desire for no major surgery. Furthermore, informed consent was obtained from the patient before the surgery. 

**Fig 2:**
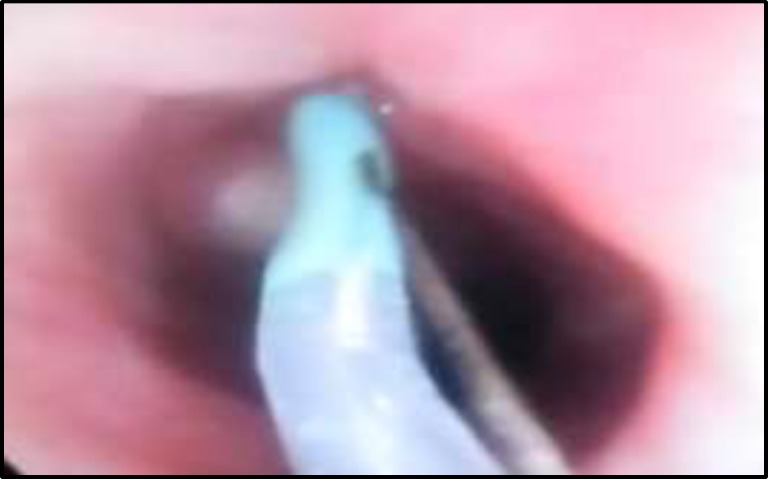
ERCP Catheter in Fistula

The case underwent an endoscopy to locate internal fistula orifice. In gastrointestinal endoscopy, a fistula was identified in the anterior wall of ​​the neopharynx (Fig.2). 

Fibrin glue was prepared from the patient’s blood sample under certain procedures. The PCF tract was endoscopically localized, and an endoscopic retrograde cholangio- pancreatography (ERCP) catheter passed through the fistula, pushed forward, and pulled backward gently to prevent injury to major vessels. The de-epithelialization of fistula orifice was performed, and the fibrin glue was endoscopically injected into the fistula tract via the catheter. Afterward, a nasogastric (NG) tube was inserted under the endoscopic view.

**Fig 3:**
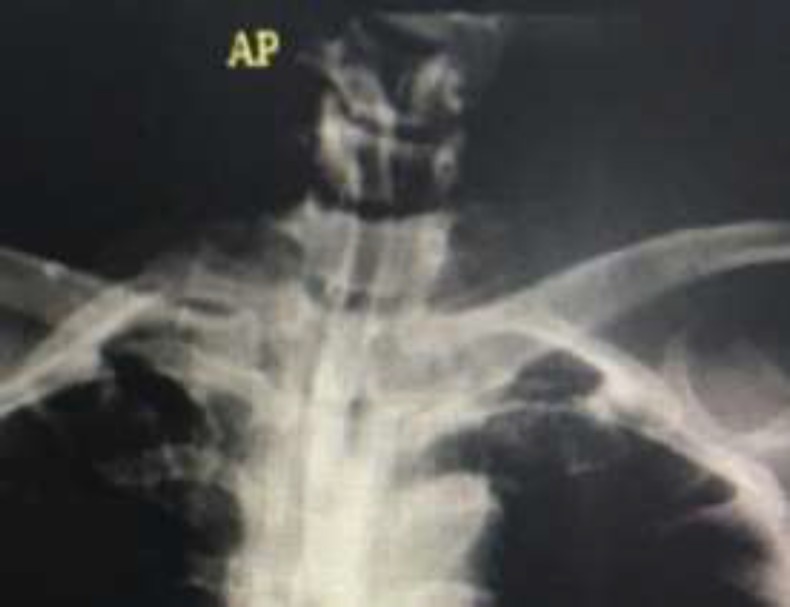
Normal passage of the contrast from neopharynx and entry into stomach one month after fibrin glue injection indicating fistula closure and patient's treatment completion

Following the administration of fibrin glue, the patient with NPO (i.e., nothing through the mouth) underwent pressure dressing and continued conservative treatment. Two weeks later, the case was discharged with an NG tube and antibiotics (oral Clindamycine 300 mg TDS). One month after the fibrin glue injection, no evidence of contrast extravasation was observed on barium swallow test, and the fistula was completely closed (Fig.3). The normal saline serum was given to the patient with a methylene blue as a surgical marker, with no evidence of leakage. When oral feeding started, the patient tolerated the pain without difficulty. The NG tube was drawn 1 month after the fibrin administration, and the patient returned to normal oral feeding without any problems.

**Fibrin glue preparation**

Two components are required to prepare fibrin glue. The first component is fibrinogen, and the second one is thrombin. Fibrinogen is prepared after the extraction of blood from the patient's vein or blood bank and a series of other steps. The second part that is thrombin is produced as a ready-to-use commercial solution, as well as being extractable from the plasma of the patient. Thrombin is diluted in distilled water and combined with aminocaproic acid as an antifibrinolytic agent. The first component is prepared by three following methods:

• The first method (plasma): Firstly, 36 ml of citrated blood from a blood bank is evenly divided into four tubes, and the tubes are centrifuged at 3000 rpm for 3 min. The plasma is then removed by pipet and combined with 1 ml of calcium chloride solution.

• The second method (plasma cryoprecipitate): Firstly, 350 ml of frozen blood in a bag (frozen at -18°C) is placed in a refrigerator at 4°C until it melts slowly. Then, the melted blood is centrifuged at 5000 rpm for 5 min. Afterward, 1mm of calcium chloride solution is added. Finally, the substance sediment in the foam bag is used as fibrinogen.

• The third method (plasma cryoprecipitate): Firstly, 36 ml of noncytarabine blood is collected in four tubes, each one containing 1 ml of 10% sodium citrated solution. Afterward, the tubes are centrifuged at 3000 rpm for 10 min. The obtained plasma is mixed with 1.3 ml of ammonium sulfate saturated solution in four silicon tubes in which fibrinogen is immediately precipitated and then centrifuged at 3000 rpm for 3 min. About 1.5 ml of white sediment is collected by siphoning and mixed with 1 ml calcium chloride.

In this study, the second method was used for the preparation of fibrin glue.

*Fibrin glue injection*

In this case, fibrin glue was injected by endoscopy. Firstly, under the intravenous sedation and left lateral decubitus position under the cardiopulmonary monitoring, the endoscope entered into neopharynx through the mouth. The location of the fistula was observed in the anterior neopharyngeal wall and 12 cm from the incisive teeth. The probe of the endoscope was fixed at the fistula location. Then, the ERCP catheter passed through the endoscope into the fistula, pushed forward, and pulled backward gently to prevent injury to major vessels. 

**Fig 4:**
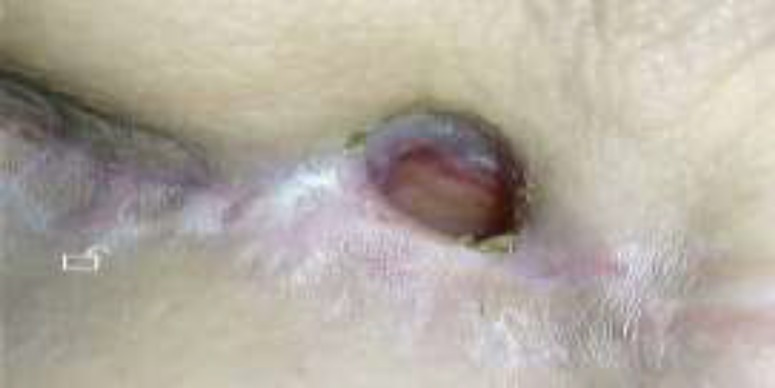
Follow up Photo

The de-epithelialization of fistula orifice was performed, and 5 cc of the fibrin glue was endoscopically injected into the fistula tract via the catheter. In addition, some fibrin glue was percutaneously injected into the outer orifice of the fistula. The patient follow-up showed that the fistula was completely closed 3 months after the administration. The NG tube was removed, and oral nutrition was successfully started. The subsequent follow-up demonstrated that the patient tolerated a soft diet and fluids without any problems (Fig.4).

## Discussion

In head and neck surgeries, the incidence of fistula after total laryngectomy is still one of the most important causes of morbidity. This complication may lead to wound infections, abscess formation, carotid rupture, and even mortality. Organ preservative treatments, such as chemoradiotherapy, are now used as the first-line treatment for laryngeal carcinoma, and total laryngectomy is considered regarding treatment failure and relapse. Tissue healing is impaired due to the degeneration of fibroblasts and collagens, reduction of mesenchymal, and migration of inflammatory cells. Therefore, the risk of developing fistula after total laryngectomy surgery increased in patients undergoing radiotherapy. 

The incidence of PCF varies widely in different studies. The reason for this variation is probably related to the type of the initial treatment of patients (i.e., surgery or chemoradiotherapy) and difference in the patient selection for the recurrent surgery. Various methods have been proposed to prevent this complication before the surgery, including preoperative prophylactic antibiotics, treatment of gastroesophageal reflux before and after surgery, and delay in starting postoperative oral feeding. However, none of these methods ensures that this complication does not occur postoperatively. 

Most of the patients with PCF are initially under supportive and conservative therapy, such as intravenous antibiotics, no permission regarding oral intake, intravenous feeding, and wound care. Many practitioners recommend that supportive care should be continued for at least 1 month. However, at the end of this period, about 30% of the patients still have fistulas and require surgical procedures to repair fistulas. Many types of surgical procedures have been introduced to close these types of fistulas. The simple suturing of the fistula, local or regional flaps, or free flaps is reported as one of these methods. There is still a risk of relapse even in some patients that ultimately close their fistulas with surgical procedures.

The first experience regarding the use of fibrin glue was its use in bleeding control by Bergel in 1909 (25). In 1944, Cronkite et al. and Tedrick and Warner were the first researchers to present the combination of fibrinogen and thrombin for the production of adhesion and use of this mixture to attach skin flaps (26,27). Fibrin glue consists of two main parts, namely a binding protein and thrombin. The binding protein contains fibrinogen, and plasma proteins include albumin, insoluble immunoglobulins, and factor XIII. Except for these two main parts, there are other components, such as calcium chloride and fibrinolysis inhibitors. When these components are combined, thrombin converts fibrinogen into fibrin and flocculation starts. With the onset of the process of flocculation, the factor XIII is converted to active factor XIII in the presence of ionized calcium and leads to the enhancement of the clot bonds. The result of all these processes is the formation of an elastic clot or fibrin clot, with adhesion and hemostatic quality. As the tissue repairs, the clot gradually lyses and is absorbed by phagocytes. 

The clot is rapidly absorbed in tissues with a good vascularization and appropriate blood supply. However, in tissues after radiotherapy, fibrosis is formed, and impaired blood supply leads to the longer remaining of the clot in the area. On the other hand, the presence of antifibrinolytic agents, such as aprotinin in the clot, prevents rapid lysis. All of these factors help the mucus edges to be joined more accurately in the presence of fibrin glue.

McCarthy et al. used fibrin glue in 1987 to prevent esophageal and anastomotic leakages. In 1989, Romeo et al. applied fibrin glue to close PCF (28,29). In a study performed by Saclarides et al. )^30^(, fibrin glue was employed after the radiotherapy of small intestinal anastomosis in 1992. In 1995, Wiseman et al. used fibrin glue for the treatment of recurrent tracheoesophageal fistula with an endoscopic method (31). In a study carried out by Ussiaet al. in 1998, fibrin glue was applied to improve the success of colon anastomosis )^32^). In addition, Scappaticci et al. utilized fibrin glue to close the bronchopulmonary fistulas with the endoscopic approach in 2000 )^33^(. 

According to the literature, Kackeret al. used fibrin glue to close end-to-end tracheal anastomosis in 2001)^34^(. Sentovich utilized fibrin glue to close the perianal fistulas in 2001(35). Based on the evidence, there is only one case regarding the use of fibrin glue in the closure of PCFs. In 2003, Wiseman et al. presented a 68-year-old African-American man with the squamous cell carcinoma of the larynx at the T2N0M0 stage )^36^(, initially treated via radiation therapy with a dose of 6840c Gy. Next year, the patient was visited for odynodysphagia and progressive weight loss, and salvage total laryngectomy and bilateral comprehensive neck dissection were performed after the observation of local relapse. Furthermore, the pectoralis major myocutaneous flap was reconstructed for a surgical defect. Two weeks after the operation, the patient was admitted due to PCFs, and the supportive care started. Despite 2 months of conservative treatment and lack of fistula closure, the patient ultimately underwent surgery to repair the fistula. During the surgery, the fistula was resected after the separation of the mucous membrane from the subcutaneous tissues and skin. The mucus edges came close to each other with absorbable sutures. Then, the area covered with 4 ml of fibrin glue, and a lining layer was provided. Afterward, the subcutaneous and cutaneous layers were sutured in separate plans. After 6 months of follow-up, the fistula was completely closed, and the patient tolerated the soft and liquid diet.

## Conclusion

To the best of our knowledge, no PCF has been treated with fibrin glue using only the endoscopic technique. Although the authors' experience in this regard is preliminary, the results of the present study showed that fibrin glue can be used as an effective way to treat chronic fistulas in head and neck surgeries.

## References

[1] Sassler AM, Esclamado RM, Wolf GT (1995). Surgery after organ preservation therapy Analysis of wound complications. Arch Otolaryngol Head Neck Surg..

[2] Cho BC, Kim M, Lee JH, Byun JS, Park JS, Baik BS (1998). Pharyngoesophageal reconstruction with a tubed free radial forearm flap. J ReconstrMicrosurg..

[3] Weber RS, Berkey BA, Forastiere A, Cooper J, Maor M, Goepfert H (2003). Outcome of salvage total laryngectomy following organ preservation therapy: the Radiation Therapy Oncology Group trial 91–11. Arch Otolaryngol Head Neck Surg..

[4] Ganly I, Patel S, Matsuo J, Singh B, Kraus D, Boyle J (2005). Postoperative complications of salvage total laryngectomy. Cancer..

[5] Sewnaik A, Keereweer S, Al-Mamgani A (2012). High complication risk of salvage surgery after chemoradiation failures. ActaOtolaryngol..

[6] Paydarfar JA, Birkmeyer NJ (2006). Complications in head and neck surgery: a meta-analysis of postlaryngectomypharyngocutaneous fistula. Arch Otolaryngol Head Neck Surg..

[7] Tsou YA, Hua CH, Lin MH, Tseng HC, Tsai MH, Shaha A (2010). Comparison of pharyngocutaneous fistula between patients followed by primary laryngopharyngectomy and salvage laryngopharyngectomy for advanced hypopharyngeal cancer. Head Neck..

[8] Erdag MA, Arslanoglu S, Onal K, Songu M, Tuylu AO (2013). Pharyngocutaneous fistula following total laryngectomy: multivariate analysis of risk factors. Eur Arch Otorhinolaryngol..

[9] Hier M, Black MJ, Lafond G (1993). Pharyngo-cutaneous fistulas after total laryngectomy: incidence, etiology and outcome analysis. J Otolaryngol.

[10] Boscolo-Rizzo P, De Cillis G, Marchiori C, Carpene S, Da Mosto MC (2008). Multivariate analysis of risk factors for pharyngocutaneous fistula after total laryngectomy. Eur Arch Otorhinolaryngol.

[11] Galli J, De Corso E, Volante M, Almadori G, Paludetti G (2005). Postlaryngectomypharyngocutaneous fistula: incidence, predisposing factors, and therapy. Otolaryngol Head Neck Surg.

[12] White HN, Golden B, Sweeny L, Carroll WR, Magnuson JS, Rosenthal EL (2012). Assessment and incidence of salivary leak following laryngectomy. Laryngoscope.

[13] Aarts MC, Rovers MM, Grau C, Grolman W, van der Heijden GJ (2011). Salvage laryngectomy after primary radiotherapy: what are prognostic factors for the development of pharyngocutaneous fistulae?. Otolaryngol Head Neck Surg.

[14] Horgan EC, Dedo HH (1979). Prevention of major and minor fistulae after laryngectomy. Laryngoscope.

[15] Lavelle RJ, Maw AR (1972). The aetiology of post-laryngectomy pharyngo-cutaneous fistulae. J Laryngol Otol.

[16] Basheeth N, O'Leary G, Sheahan P (2013). Pharyngocutaneous fistula after salvage laryngectomy: impact of interval between radiotherapy and surgery, and performance of bilateral neck dissection. Head Neck.

[17] Chee N, Siow JK (1999). Pharyngocutaneous fistula after laryngectomy-incidence, predisposing factors and outcome. Singapore Med J.

[18] Markou KD, Vlachtsis KC, Nikolaou AC, Petridis DG, Kouloulas AI, Daniilidis IC (2004). Incidence and predisposing factors of pharyngocutaneous fistula formation after total laryngectomy Is there a relationship with tumor recurrence?. Eur Arch Otorhinolaryngol.

[19] Patel UA, Moore BA, Wax M, Rosenthal E, Sweeny L, Militsakh ON (2013). Impact of pharyngeal closure technique on fistula after salvage laryngectomy. JAMA Otolaryngol Head Neck Surg..

[20] Qureshi SS, Chaturvedi P, Pai PS (2005). A prospective study of pharyngocutaneous fistulas following total laryngectomy. J Cancer Res Ther.

[21] Davis RK, Vincent ME, Shapshay SM, Strong MS (1982). The anatomy and complications of “T” versus vertical closure of the hypopharynx after laryngectomy. Laryngoscope.

[22] Cavalot AL, Gervasio CF, Nazionale G, Albera R, Bussi M, Staffieri A (2000). Pharyngocutaneous fistula as a complication of total laryngectomy: review of the literature and analysis of case records. Otolaryngol Head Neck Surg..

[23] Hanasono MM, Lin D, Wax MK, Rosenthal EL (2012). Closure of laryngectomy defects in the age of chemoradiation therapy. Head Neck..

[24] Paleri V, Drinnan M, van den Brekel MW, Hinni ML, Bradley PJ, Wolf GT (2014). Vascularised tissue to reduce fistula following salvage total laryngectomy: a systematic review. Laryngoscope..

[25] Bergel S (1909). Uber Wirkungen des Fibrins. Dtsch Med Wochenschr.

[26] Cronkite EP, Lozner EL, Deaver JM (1944). Use of thrombin and fibrinogen in skin grafting. JAMA.

[27] Tedrick RT, Warner ED (1944). Fibrin fixation of skin transplants. Surgery.

[28] McCarthy PM, Trastek VF, Schaff HV, Weiland LH, Bernatz PE, Payne WS (1987). Esophagogastric anastomoses: The value of fibrin glue in preventing leakage. J Thorac Cardiovasc Surg.

[29] Romeo G (1989). Applications of tissucol in larynx, tracheaand neck surgery. Rev LaryngolOtol Rhinol (Bord).

[30] Saclarides TJ, Woodard DO, Bapna M, Economou SG (1992). Fibringlue improves the healing of irradiated bowel anastomoses. Dis Colon Rectum.

[31] Wiseman NE (1995). Endoscopic closure of recurrent tracheoesophagealfistula using Tisseel. J PediatrSurg.

[32] Ussia G, Cuccomarino S, Ravo B, Galletti G (1998). Combined laparoendoscopic colon resection and anastomosis usingthe “no touch technique” and fibrin glue. An experimentalstudy. SurgEndosc.

[33] Scappaticci E, Ardissone F, Ruffini E, Baldi S, Revello F, Coni F (2000). As originally published in 1994: Postoperative bronchopleural fistula: Endoscopic closure in 12 patients. Updated in2000. Ann ThoracSurg.

[34] Kacker A, Huo J (2001). Reinforcement of an end-to-endtracheal resection anastomosis with fibrin glue: A casereport. Ear Nose Throat J.

[35] Sentovich SM (2001). Fibrin glue for all anal fistulas. J GastrointestSurg.

[36] Wiseman S, Hicks W, LoreeTh, Al-kasspooles M, Rigual N (2002). Fibrin Glue–Reinforced Closure of PostlaryngectomyPharyngocutaneous Fistula. Am J Otolaryngol.

